# Melanoma of the Scalp and Neck: A Population-Based Analysis of Survival and Treatment Patterns

**DOI:** 10.3390/cancers14246052

**Published:** 2022-12-08

**Authors:** Matteo Scampa, Vladimir Mégevand, Juan A. Viscardi, Salvatore Giordano, Daniel F. Kalbermatten, Carlo M. Oranges

**Affiliations:** 1 Department of Plastic, Reconstructive, and Aesthetic Surgery, Geneva University Hospitals, Geneva University, 1205 Geneva, Switzerland; 2 Department of Plastic Surgery, Guy’s and St Thomas’ NHS Foundation Trust, St Thomas’ Hospital, London SE1 7EH, UK; 3 Department of Plastic Surgery, Turku University Hospital, University of Turku, 20500 Turku, Finland

**Keywords:** melanoma, scalp, neck, SEER, survival, surgical margin, epidemiology

## Abstract

**Simple Summary:**

Surgery remains the mainstem of treatment. Scalp melanoma is reputed for carrying a worse prognosis than other locations. Our aim is to explore how demographics, clinic-pathologic factors and surgical margins affect overall survival. We identified male gender, tumour ulceration, high mitotic rate or nodular subtype as negative prognostic factor of survival. No survival benefit with margins over 2 cm was seen. For a Breslow thickness between 1.1 and 2 mm, for which current National Comprehensive Cancer Network recommendations allow a flexibility to choose margins between 1 and 2 cm, no significant difference was seen between <1 cm margins and 1 to 2 cm margins. According to our results, a more conservative approach with 1 cm margin might not impact survival.

**Abstract:**

Introduction: Melanoma is an aggressive skin cancer. Large demographic and clinic-pathologic studies are required to identify variations of tumour behavior. The aim of our study was to offer updated epidemiologic data on the scalp and neck melanoma with an overall survival analysis. Method: The SEER database was searched for all scalp and neck melanoma in adult patients between 2000 and 2019. Demographic and clinic-pathologic variables were described. Their impact on overall survival was assessed with the log-rank test after Kaplan–Meier model. A multivariable cox-regression was conducted to identify predictors of decreased survival. A *p*-value of <0.005 was considered statistically significant. Results: 20,728 Melanomas of the scalp and neck were identified. Mean age was 62.5 years. Gender ratio was 76.3% males. 79% of the tumours were localized at diagnosis. Increasing age, male gender, tumour ulceration, high mitotic rate or nodular subtype were independent prognostic factors of decreased overall survival. Surgery with less than 1 cm margin is associated with the best overall survival in this cohort. No significant difference in OS was seen between less than 1 cm and 1 to 2 cm margins. Conclusion: Knowledge of negative prognostic factors might help identify subgroups at risk and adapt their oncologic treatment.

## 1. Introduction

Melanoma is an aggressive cutaneous neoplasm originating from melanocytes. Worldwide, its incidence is rising, and the disease is becoming a real burden [1,2]. Despite prevention campaigns and early detection protocols, melanoma strongly impacts national healthcare and generates important costs [3,4]. UV exposure is an important risk factor for melanoma. Scalp and neck are sun exposed areas subject to melanoma. Scalp melanoma is believed to be more aggressive due to a higher Breslow thickness, a higher mitotic rate and a high propensity to peri-neural invasion, resulting in lower survival outcomes [5,6,7,8,9].

Melanoma treatment in the head and neck is based on a multi-disciplinary approach, where plastic surgeons, oncologic surgeons, and head and neck surgeons are responsible for wide resection and sentinel lymph node biopsy (SLNB). Mohs surgeons are also involved when there is no need for SLNB. However, plastic surgery plays a prominent role in performing the surgical removal of melanoma in the scalp/neck, given the frequent need of reconstructive procedures for both functional and aesthetic purposes. Melanoma surgery requires complete tumour resection with safety margins varying according to AJCC staging [10]. Currently the NCCN recommends 0.5 to 1 cm margins for in situ tumours, 1 cm margins for Breslow ≤1 mm, 1 to 2 cm margins for a Breslow between 1 and 2 mm, and 2 cm margins for Breslow thickness between 2 and 4 mm and over 4 mm [11]. Large defects are often created by wide margin resection and in the scalp and neck they can induce real reconstructive challenges, especially if surgery is associated with radiation therapy. A more conservative approach in the head and neck is currently under investigation to avoid surgical morbidity [12]. To optimize patient care and oncological outcomes, it is of primordial importance to understand the tumour’s behavior and to know how different variables can impact survival. Large population studies are required to offer strong evidence with high statistical power. The Surveillance, Epidemiology and End Results (SEER) is a program of the National Cancer Institute (NCI) collecting cancer data through registries representative of the US population.

The aim of our current study is to analyze demographic and clinic-pathological data of the scalp and neck melanoma to determine if narrower margin resection can offer similar overall survival than wide margins.

## 2. Materials and Methods

Patient selection: Seventeen SEER registries were searched for all cases of melanoma of the skin of scalp and neck diagnosed between 2000 and 2019 in adult patients (18 years old and more), using ICD-O3 code 8720 to 8790 and primary site localization code C44.4. The follow-up cut-off data was the 31 December 2019.

Variable selection: Demographic, clinical-pathological and treatment variables were extracted from the SEER database. Variables containing multiples values were allied in subgroups such as the surgery variable in which subgroups consisted in no surgery, tumour resection/destruction without margin, less than 1 cm margins surgery, Mohs surgery (independent of the margins), 1 to 2 cm margins surgery and more than 2 cm margins. In some cases, margins were described only as more than 1 cm, meaning they were categorized in the 1 to 2 cm subgroup. Regarding age related survival analysis, patients were arbitrary split in 3 categories according to their age: 18 to 49, 50 to 69 and 70 or more. This division aimed at identifying survival differences between young, middle aged and older patients. Breslow analysis was sub-divided into 4 categories: less or equal to 1 mm, 1.1 to 2 mm, 2.1 to 4 mm and more than 4 mm. This subdivision follows AJCC classification and NCCN recommendations for surgical margins according to Breslow thickness [10,11]. Breslow scores over 9.8 mm were reported as 9.8 mm in the database. Breslow scores reported as Greater than 0.0 mm and less than or equal to 0.1 mm were reported as 0.1 mm. Breslow thickness was reported only for cases diagnosed in 2010 or later. Mitotic rate analysis was reported from 1 to 10, then as more than 11 mitosis per square mm. We further subdivided it into 3 categories according to NCCN risk stratification: Less or equal to 2 mitosis per square mm, 3 to 10 mitosis per square mm, 11 or more mitosis per square mm [11]. Tumor stage was based on the SEER summary stage variable as the AJCC stage was inconsistently reported, despite being validated for prognostic value [10]. Localized disease was defined as a tumor confined to the epidermis and dermis corresponding to AJCC stage I and II, while regional diseases required subcutaneous invasion, in transit metastases, satellite lesions or/and positive regional lymph nodes (AJCC stage III). Distant disease encompassed all metastases, including distant lymph node groups (AJCC stage IV). Regarding histologic subtype analysis, diagnosis was described using ICD-O3 codes in the SEER database. These codes were used for subtype analysis. Furthermore, cases coded as melanoma without specifying the histological subtype were coded in the SEER database as 8720/3 Malignant melanoma, Not otherwise specified (NOS).

Statistical analysis: Data was extracted from SEER*stat v.8.4.0.1 and processed through analytical software IBM SPSS version 27 (IBM, Armonk, NY, USA). Overall survival (OS) analysis of study population was conducted using the Kaplan–Meier method and survival curves were created. Univariable comparison was done with the log rank Test. Variables values with less than 100 occurrences were not included in survival analysis because they would lack statistical power. For univariable analysis of OS according to surgical therapy, we conducted a subgroup analysis where survival curves were stratified by Breslow thickness. A multivariable analysis using a cox-regression model was run to identify independent prognostic factors. The proportional hazard assumption was considered as met, but not tested before running the model. Variables analysed were adjusted for confounders and moderators. A *p*-value < 0.005 was considered statistically significant.

## 3. Results

The selection criteria allowed to identify 20,728 melanomas of the scalp and neck. Most of the cohort were males (76.3%) with a mean age of 62.5 years. Demographic results of the population are presented in Table 1. Seventeen different histological subtypes of melanoma were described. Other subtypes included: 8722/3: Balloon cell melanoma; 8723/3: Malignant melanoma, regressing; 8730/3: Amelanotic melanoma; 8740/3: Malignant melanoma in junctional nevus; 8744/3: Acral lentiginous melanoma, malignant; 8761/3: Malignant melanoma in giant pigmented nevus; 8770/3: Mixed epithelioid and spindle cell melanoma; 8771/3: Epithelioid cell melanoma; 8773/3: Spindle cell melanoma, type A; 8780/3: Blue nevus, malignant. Breslow was reported inconsistently through the cases with a mean value of 1.49 mm, standard deviation 2.09 mm. The value of Breslow was reported for only 10,124 cases.

**Figure 1:**
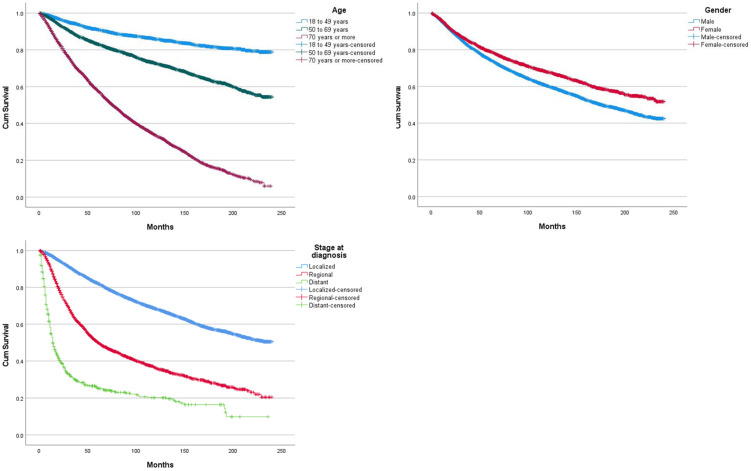
Overall survival curves, according to age, gender, and stage at diagnosis.

**Figure 2:**
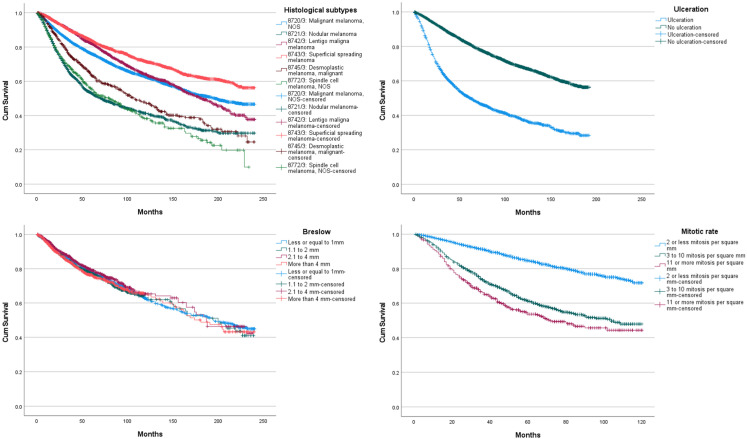
Overall survival curves, according to histological subtype, presence of ulceration, Breslow thickness, and mitotic rate.

Mean overall survival was 156.2 months (SD 0.799), median overall survival was 194 months (SD 4.067). One, 3 and 5 years OS rate was, respectively, 94.9%, 83.7% and 75.9%. Increasing age was significantly associated with lower OS (*p* < 0.005; Figure 1). Males OS was lower than females (*p* < 0.005). More advanced disease was associated with a significantly lower OS (*p* < 0.005).When assessing survival according to histological subtypes, the difference between all subtypes of survival probability is statistically significant except between 8720/3 Malignant melanoma, NOS and 8742/3 Lentigo maligna melanoma.(Figure 2) Superficial spreading melanoma (8743/3) offers the best OS (mean 177.4 m 95% CI [174.5–180.4]) while 8721/3 Nodular melanoma (mean 112 m 95% CI [106.7–117.2]) and 8772/3 Spindle cell melanoma (mean 104 m 95% CI [94.6–113.5]) have the worst OS. No significant difference in OS is between 8721/3 Nodular melanoma and 8772/3 Spindle cell melanoma, NOS. No significant difference in OS was seen with increasing Breslow depth. (*p* > 0.170) The presence of ulceration in the tumour is associated with a significantly lower OS (mean 91.4 m 95% CI [88–94.9]; *p* < 0.005) compared to tumour without ulceration. (143.4 m 95% CI [142–144.8]; *p* < 0.005) Increasing mitotic rate was associated with lower OS in univariable analysis. (*p* < 0.005).

**Figure 3:**
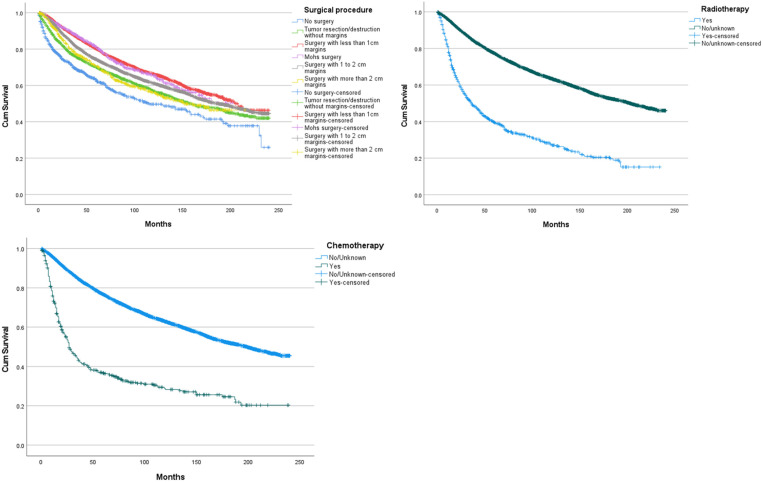
Overall survival curves, according to surgical procedure, radiotherapy, and chemotherapy.

When assessing how OS was impacted between different surgical strategies without Breslow stratification, no significant difference was seen between surgery with <1 cm margins and Mohs surgery (*p* = 0.622; Figure 3), between Mohs surgery and surgery with 1 to 2 cm margins (*p* = 0.032), between tumour resection/destruction without margins and surgery with more than 2 cm margins (*p* = 0.760), surgery with more than 2 cm margins and surgery with 1 to 2 cm margins (*p* = 0.016). The use of radiotherapy was associated with a significantly lower OS (mean 78.8 m 95% CI [71.9–81.6]; *p* < 0.005) compared to those who were categorized under no/unknown. Despite less than 500 occurrences, chemotherapy was included in survival analysis as it represents an important treatment modality. The use of chemotherapy was associated with a significantly lower OS (mean 81.4 m 95% CI [71.6–91.2]; *p* < 0.005) compared to those who were categorized under no/unknown.

**Figure 4:**
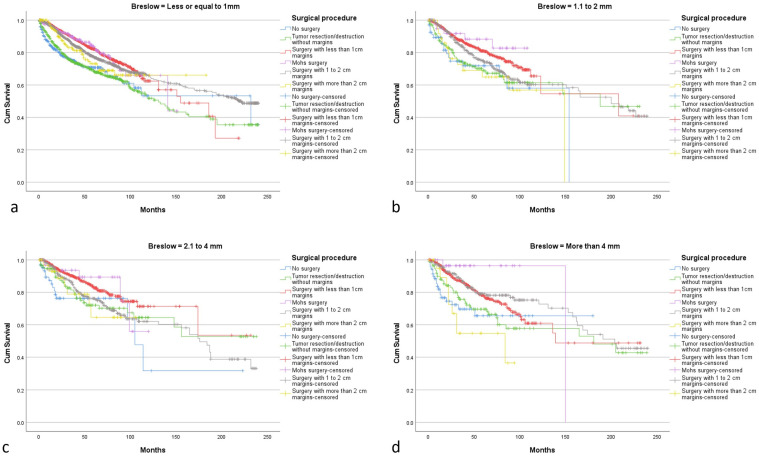
(**a**–**d**) Overall survival curves according to surgical procedure, stratified by Breslow thickness.

In subgroup analysis: Surgery distribution according to Breslow thickness is described in Table 2. For a Breslow ≤ 1 mm best OS was achieved with surgery with 1 to 2 cm margins (mean 163.4 m 95% CI [157.1–169.8]). (Figure 4a) However, difference with less than 1 cm margins surgery, Mohs surgery and, more than 2 cm margins surgery was not statistically significant. For a Breslow between 1.1 to 2 mm, surgery with less than 1 cm margins (mean 158 m 95% CI [133.6–182.4]) and surgery with 1 to 2 cm margins (mean 154.7 m 95% CI [141.9–167.5]) were associated with the best OS. (Figure 4b) No statistically significant difference was found between them. For a Breslow between 2.1 and 4 mm, surgery with less than 1 cm margins was associated with the highest OS (mean 170.7 m 95% CI [150–191.4]). (Figure 4c) OS difference with all the other surgeries was not statistically significant. For a Breslow over 4 mm, surgery with 1 to 2 cm margins was associated with the highest OS (mean 168.6 m 95% CI [152.4–184.8]). (Figure 4d) It was significantly better than surgery with more than 2 cm margins and no surgery (*p* < 0.005), no significant difference with other surgical modalities was seen.

In multivariable analysis, increasing age, male gender, nodular histologic subtype, regional and distant disease, the use of radiotherapy, the use of chemotherapy, increasing mitotic rate and the presence of an ulceration on the primary tumour were identified as independent prognostic factors of decreased OS. (Table 3) Breslow thickness and type of surgical resection did not affect OS in multivariable analysis.

## 4. Discussion

This study includes 20,728 melanomas of the scalp and neck region and represents the largest population in a single study focusing on this area. A study on the SEER database assessing the OS of scalp and neck melanoma and comparing it with other regions has been published in 2008 [13]. This study was based only on 13 registries and included 3271 cases of scalp and neck melanoma. Scalp and neck melanomas are known to be associated with a worse prognosis than other locations [9,14].

In this cohort where most of the tumors were localized, OS was considerably low with 75.9% of the patients surviving after 5 years.

Population age was slightly higher with a mean age of 62.5 years old compared to the previous SEER study focusing on the scalp and neck region (mean age 58.6 years old) [13]. This result can be explained by an aging population. It stays in line with other studies, reporting a mean age at diagnosis between 60 and 70 years old for head and neck melanomas [5,15]. However, it has been previously identified that head and neck melanomas tend to develop at an older age than in other locations [16]. In the scalp and neck region, increased age was associated with a lower OS. Age strongly impacts OS because any cause of death is accounted even though it is not related to tumoral progression. This phenomenon can be partially related to a higher susceptibility to adverse effects resulting from oncologic treatments in older populations. In multivariable analysis, an age of 70 years or more increased the odds ratio of dying up to 6.99.

Interestingly, in the scalp and neck region, males are more affected than females with 76% of males being affected. This trend has already been described in the head and neck region but seems to be stronger in the scalp and neck [8,13,16,17]. As Licata et al., suggested in their review, this might be linked to hair density, protecting the skin of the scalp and neck from UV exposure [18]. Gender also impacts survival with lower OS for males in univariable and multivariable analysis. Bald scalps have been found to have deeper Breslow thickness than hairy scalp, potentially explaining the lower OS in males [19]. Furthermore, balding increases with age, meaning that exposed populations are the elderly, which are less susceptible to present to a dermatologist or practician, resulting in late diagnosis. De Vries et al., explained this observation by a diagnosis at an earlier stage in females [20].

In the scalp and neck region, Caucasian ethnicity remains the most affected one due to correlation with light skin phototype and melanoma development.

Regarding histologic subtypes, most of the cases were categorized as malignant melanoma without more details (44.7%). The proportion of superficial spreading melanoma, also called pagetoid melanoma remains high (25.8%), but still lower than SEER studies where all locations are reported [21]. It has been reported in literature as the most frequent histologic subtype [22]. Interestingly, the proportion of lentigo maligna melanoma and nodular melanoma is higher than in Pollack et al.’s study [21]. Interpretation of histologic subtype distribution must be cautious given that most of the cases are classified as NOS. In univariable analysis, nodular melanoma was found to have the lower OS. This is mainly due to its vertical growth pattern with a rapidly increasing Breslow depth [23]. Worst OS is confirmed for nodular melanoma also in multivariable analysis when adjusted to age and sex.

Most cases were diagnosed at a localized stage (79%). In situ melanoma were extremely scarce. It can be explained by an under-report of in situ tumours, or by late diagnosis in this region. The proportion of regional and distant diseases is similar to Pollack et al.’s study where all skin locations were assessed [21]. In univariable and multivariable analysis, tumoural dissemination to lymph nodes or distant site was associated with lower OS. Regional lymph node positivity has been previously identified as a negative predictor of survival. Furthermore, a study described LN metastasis as a strong predictor of low OS specifically in scalp melanoma [24]. Sentinel lymph node biopsy maintains an important role in tumour staging [11,25,26].

Breslow depth was reported in half of the patients with a mean value of 1.49 mm. Precaution should be taken when interpreting those results as unreliable report of tumour thickness in the SEER database has been described and data of patients diagnosed before 2010 was not reported [27]. This is deeper than values reported in studies assessing overall locations [13]. This difference has been already described in various studies and might explain the worst OS [5,15]. However, in univariable and multivariable analyses no significant difference in OS is seen. Breslow depth defines surgical guidelines; therefore, a deeper Breslow might induce more aggressive treatment with wider resection margins, explaining a potential equivalent OS. On the other hand, Breslow thickness has been identified in many studies as a prognostic factor of worse survival, so it would be expected to alter OS in our cohort [28,29]. The absence of Breslow thickness report for half of the study population (before 2010) and may be a limitation in the study design.

Ulceration rate and mitotic rate were both higher in our cohort compared to studies assessing overall distribution of melanoma [13,30]. Increasing mitotic rate and presence of ulceration were both associated with lower OS in univariable and multivariable analysis in our study. Furthermore, some studies already identified scalp melanoma as presenting with higher mitotic rates and higher proportion of ulcerated tumours [5,31]. This might also explain why scalp melanoma presents a worst prognosis than when located in other regions of the body [9]. In the latest revision of AJCC classification, mitotic rate was removed as a criterion for staging [32]. However, in our study results support the impact of mitotic rate on prognosis. Kashani-Sabet et al., observed similar results and advocates in favor of mitotic rate reincorporation in the T category [33].

Surgery with less than 1 cm margins was the most frequent therapeutic modality in our cohort, followed by surgery with 1 to 2 cm margins. Wide excision with more than 2 cm margins remained rare in our cohort, so did Mohs surgery. Surgical margin selection is mainly based on the Breslow depth and the presence of tumour characteristics associated with low OS such as ulceration, nodular subtype, etc. [18]. When assessing the whole cohort, surgery with less than 1 cm margins offered the best OS, compared to wider resection margins. No significant difference of survival was seen between less than 1 cm margins and Mohs surgery. No significant difference of survival was seen between 1 to 2 cm margins and wider than 2 cm margins. It can be mainly explained by less than 1 cm margin resections being performed in tumors with low-risk characteristics (shallow Breslow, no ulceration). According to NCCN guidelines, 1 cm margins are recommended in tumours with a Breslow thickness of 1 mm or less. For Breslow between 1.1 and 2 mm, recommendations are 1 to 2 cm margins. If we assess the subgroup analysis, no significant difference was seen between less than 1 cm margins and 1 to 2 cm margins for all Breslow thickness strata. Those results support current NCCN guidelines. This applies in particular for a Breslow between 1.1 and 2 mm where recommendations suggest margins between 1 and 2 cm. Koskivuo et al., suggested 1 cm margins might be sufficient in this case [34]. As wider excision can lead to important defects, necessitating more complex reconstructive procedures, further studies should be conducted to assess feasibility of 1 cm margins resection in the scalp for this Breslow thickness range.

Aggressive treatment is done in more aggressive tumours, explaining the lower OS seen in more than 2 cm margins surgery in the overall cohort. In subgroup analysis, for a Breslow of more than 4 mm, 1 to 2 cm margins resection offered a significantly better OS than more than 2 cm margins. This illustrates why recommendations limit to 2 cm security margins, as wider margins don’t improve survival [12]. In this study the lower OS with wider margins can also be associated with more advanced tumours as seen in subgroup analysis where more than 2 cm margins had significantly lower OS in the subgroup analysis with a Breslow of more than 4 mm compared to 1 and 1 to 2 cm margins. The absence of difference between Mohs surgery and <1 cm margins or 1 to 2 cm margins in the overall cohort can be explained because Mohs surgery value include patients with >1 cm or <1 cm margins without distinction between them. Debate around the optimal margin for tumour resection subsists as described in Hanna et al.’s meta-analysis [12,35]. Wider margins result in the necessity of a reconstructive strategy. Scalp and neck differ significantly in terms of reconstructive process. The Neck region offers laxity and high mobility of the skin allowing direct closure, whereas the scalp area presents with a low mobility due to adherence to the galea requiring local tissue rearrangements or flaps to cover the defect. Debate subsists on the optimal timing of reconstruction. Previously negative margins had to be confirmed to proceed with the reconstruction, while current evidence suggests that immediate reconstruction is feasible and safe [36]. In order, to avoid complex reconstructive procedures, the use of smaller surgical margins should be assessed. In this study, no OS advantage was seen with more than 2 cm margins, advocating in favor of current recommendations that limit excision to 2 cm margins. Another SEER study suggested there might not be a difference in disease specific survival between 1–2 cm and more than 2 cm margins after stratification by T stage [37]. The use of radiotherapy and chemotherapy remains scarce in our cohort (<5%). Indications for radiotherapy are limited in melanoma treatment. It is mainly used for metastatic disease in combination with immunotherapy. Primary site radiation is limited due to the proximity with the central nervous system and the relative radio-resistance of melanoma cells requiring high doses [38]. Chemotherapy is not commonly used, due to the advent of Immunotherapy that is commonly used in melanoma multimodal approach [39,40]. A main limitation of this study is the absence of data regarding immunotherapy in the SEER database. Both radiotherapy and chemotherapy were associated with low OS in our cohort. It can be explained by their limited indications to advanced tumor stages only.

This study offers updated demographic and clinic-pathological data on the scalp and neck melanoma. Predictors of decreased overall survival are increasing age, male gender, presence of ulceration or high mitotic rate, histologic subtypes such as nodular melanoma and regional or distant invasion. Surgery with less than 1 cm margins seems to offer the best OS, most likely as it is conducted in cases presenting with less advanced tumors. Knowledge and understanding of all these factors predicting worse survival might guide the practician towards a rather more patient-specific approach orientating surgical therapy towards less invasive resections and allowing to avoid complex reconstructive procedures therefore limiting surgical morbidity.

Despite those insights, this study suffers from several limitations linked to the SEER database. The variables reported are sometimes incomplete or not sufficiently precise. Furthermore, scalp and neck are reported as a single entity and those 2 regions with different behavior cannot be assessed independently. A possible limitation is the use of OS instead of net survival. We chose this method of survival analysis as it is representative of real-life conditions, however, interpretation of the impact of certain variables on survival might be biased by confounders.

For margins assessment, they are reported as ranges, without individual values limiting analysis. Furthermore, the incompleteness of AJCC stage report limit the analysis to SEER summary stage variable based on AJCC values but combines AJCC stage 1 and 2.

## 5. Conclusions

Melanoma of the scalp and neck is a deadly tumour mainly affecting white-colored males in their sixties. Increasing age, male gender, tumour ulceration, high mitotic rate or nodular subtype at the histopathological analysis suggest a worst overall survival. In our study, no significant differences were observed in terms of overall survival between tumours resected with less than 1 cm margins and those with 1 to 2 cm margins, even after stratification by Breslow thickness. In case of a Breslow thickness standing between 1 and 2 mm, where the official recommendations suggest a resection range of 1–2 cm, these results could guide the surgeon towards a less invasive resection, limited to 1 cm margins. The use of smaller margins might as well ease the reconstructive approach after tumoral resection.

## Figures and Tables

**Table 1:** Study population.

Variable	N (%)
Total	20,728 (100)
**Age**	
Mean (SD)	62.5 (16.5)
18 to 49 years	4449 (21.5)
50 to 69 years	8546 (41.2)
70 years or more	7733 (37.3)
**Gender**	
Male	15,814 (76.3)
Female	4914 (23.7)
**Histologic subtype**	
8720/3 Malignant melanoma, NOS	9259 (44.4)
8721/3 Nodular melanoma	2000 (9.6)
8742/3 Lentigo maligna melanoma	2570 (12.4)
8743/3 Superficial spreading melanoma	5351 (25.8)
8745/3 Desmoplastic melanoma, malignant	636 (3.1)
8772/3 Spindle cell melanoma, NOS	483 (2.3)
Other subtypes	429 (2.1)
**Race**	
White	19,823 (95.6)
Black	55 (0.3)
Asian/pacific islander	87 (0.4)
American Indian/Alaska Native	47 (0.2)
Unknown	716 (3.5)
**Stage**	
In situ	7 (<1)
Localized	16,365 (79)
Regional	2885 (13.9)
Distant	599 (2.9)
Unknown	872 (4.2)
**Surgical procedure**	
No surgery	1053 (5.1)
Tumor resection/destruction without margin	2351 (11.3)
Mohs surgery	686 (3.3)
Surgery with <1 cm margins	9326 (45)
Surgery with 1 to 2 cm margins	6563 (31.7)
Surgery with >2 cm margins	600 (2.9)
Unknown	149 (0.7)
**Radiotherapy**	
Yes	806 (3.9)
No/unknown	19,922 (96.1)
**Chemotherapy**	
Yes	434 (2.1)
No/unknown	20,294 (97.9)
**Breslow**	
N = 10,124	
Mean (SD)	1.49 mm (2.09)
**Ulceration**	
Yes	2377 (11.5)
No	11,937 (57.6)
Unknown	6414 (30.9)
**Mitotic rate (mitosis per mm^2^)**	
2 or less	4820 (23.3)
3 to 10	1645 (7.9)
11 or more	503 (2.4)
Unknown	6968 (33.6)
**Variable**	N (%)

**Table 2:** Surgical procedures distribution according to Breslow thickness.

	Breslow Thickness			
Surgical Procedure	≤1 mm N = 6451	1.1–2 mm N = 1370	2.1–4 mm N = 1121	>4 mm N = 978
No surgery	416	68	66	70
Tumor resection/destruction without margins	659	134	115	112
Mohs surgery	254	45	35	32
Surgery with <1 cm margins	3434	718	599	528
Surgery with 1–2 cm margins	1529	367	274	214
Surgery with >2 cm margins	159	38	32	22

**Table 3:** Multivariable cox-regression of overall survival.

Variables	Exp(B)	95% CI	*p*-Value
**Age ^a^**			
18 to 49 years	ref		
50 to 69 years	2.142	1.947–2.358	<0.005
70 years or more	6.982	6.368–7.654	<0.005
**Gender ^b^**			
Male	ref		
Female	0.897	0.844–0.954	<0.005
**Histologic subtype ^c^**			
8720/3 Malignant melanoma, NOS	ref		
8721/3 Nodular melanoma	1.879	1.746–2.022	<0.005
8742/3 Lentigo maligna melanoma	0.709	0.653–0.770	<0.005
8743/3 Superficial spreading melanoma	0.815	0.761–0.872	<0.005
8745/3 Desmoplastic melanoma, malignant 8772/3 Spindle cell melanoma, NOS	1.177 1.480	1.039–1.333 1.299–1.685	0.010 <0.005
**Stage ^d^**			
Localized	ref		
Regional	1.856	1. 595–2.160	<0.005
Distant	4.228	3.310–5.401	<0.005
**Surgical procedure ^e^**			
No surgery	ref		
Tumor resection/destruction without margin	0.756	0.662–0.864	<0.005
Surgery with <1 cm margins	0.568	0.501–0.642	<0.005
Mohs surgery	0.606	0.483–0.711	<0.005
Surgery with 1 to 2 cm margins	0.654	0.578–0.740	<0.005
Surgery with >2 cm margins	0.621	0.519–0.741	<0.005
**Radiotherapy ^f^**			
No/unknown	ref		
Yes	1.351	1.217–1.500	<0.005
**Chemotherapy ^g^**			
No/unknown	ref		
Yes	1.514	1.323–1.732	<0.005
**Breslow thickness ^h^**			
≤1 mm	ref		
1.1–2 mm	1.043	0.919–1.183	0.515
2.1–4 mm	0.985	0.854–1.135	0.834
>4 mm	1.031	0.891–1.192	0.684
**Ulceration ^h^**			
No	ref		
Yes	2.085	1.940–2.241	<0.005
**Mitotic rate (mitosis per mm^2^) ^h^**			
2 or less	ref		
3 to 10	2.104	1.875–2.360	<0.005
11 or more	2.346	1.988–2.768	<0.005

^a^ Adjusted for sex, histological subtype. ^b^ Adjusted for age, histological subtype. ^c^ Adjusted for age, sex. ^d^ Adjusted for age, sex, histological subtype, Breslow, ulcer, mitotic rate. ^e^ Adjusted for age, sex, histological subtype, stage, radiotherapy, chemotherapy. ^f^ Adjusted for age, sex, histological subtype, stage, surgery, chemotherapy. ^g^ Adjusted for age, sex, histological subtype, stage, surgery, radiotherapy. ^h^ Adjusted for age, sex, histological subtype.

## Data Availability

Raw data available on the SEER program https://seer.cancer.gov/ (accessed on 10 August 2022).
